# Psammophytes *Alyssum desertorum* Stapf and *Secale sylvestre* Host Are Sensitive to Soil Flooding

**DOI:** 10.3390/plants13030413

**Published:** 2024-01-30

**Authors:** Elizabeth Kordyum, Yuri Akimov, Oleksandr Polishchuk, Ihor Panas, Sergiy Stepanov, Liudmyla Kozeko

**Affiliations:** 1 M.G. Kholodny Institute of Botany, National Academy of Sciences of Ukraine, 2 Tereschenkivska Str., 01024 Kyiv, Ukrainemrpolishchuk@gmail.com (O.P.); serhiy1986@ukr.net (S.S.); liudmyla.kozeko@gmail.com (L.K.); 2 Palladin Institute of Biochemistry, National Academy of Sciences of Ukraine, 9 Leontovicha Str., 01030 Kyiv, Ukraine; i.d.panas@gmail.com

**Keywords:** ADH, chlorophyll *a* fluorescence, ethylene, HSP70, photosynthetic apparatus, soil flooding, stress tolerance, ultrastructure

## Abstract

While morphological and functional traits enable hydrophytes to survive under waterlogging and partial or complete submergence, the data on responses of psammophytes—sand plants—to flooding are very limited. We analyzed the effect of 5- and 10-day soil flooding on the photosynthetic apparatus and the synthesis of alcohol dehydrogenase (ADH), heat shock proteins 70 (HSP70), and ethylene in seedlings of psammophytes *Alyssum desertorum* and *Secale sylvestre* using electron microscopy, chlorophyll *a* fluorescence induction, and biochemical methods. It was found that seedlings growing under soil flooding differed from those growing in stationary conditions with such traits as chloroplast ultrastructure, pigment content, chlorophyll fluorescence induction, and the dynamics of ADH, HSP, and ethylene synthesis. Although flooding caused no apparent damage to the photosynthetic apparatus in all the variants, a significant decrease in total photosynthesis efficiency was observed in both studied plants, as indicated by decreased values of φR0 and PI_ABS,total_. More noticeable upregulation of ADH in *S. sylvestre*, as well as increasing HSP70 level and more intensive ethylene emission in *A. desertorum*, indicate species-specific differences in these traits in response to short-term soil flooding. Meanwhile, the absence of systemic anaerobic metabolic adaptation to prolonged hypoxia causes plant death.

## 1. Introduction

Forecasts of climate global changes—warmer temperatures, flooding, and drought—focus their attention on the problem of how plants, as sessile organisms, survive in the varying environment, including adverse changes in ecological factors. Soil flooding quickly depletes oxygen, which, in water, has a low solubility and diffusion rate [1], alters plant metabolism, and inhibits aerobic respiration. Hereupon, soil flooding is one of the remarkable abiotic factors that negatively affects growth of the most terrestrial plants up to death [2,3,4]. The sensitivity and tolerance of plants to flooding significantly depend on the ecotype, the duration of flooding, and running or stagnant flood water [2]. The structural, physiological, and metabolic features that enable hydrophytes—an ecological group of plants growing in soil saturated with water (waterlogging) or in water (submerged rooted or floating plants and aerial–aquatic plants)—to survive and reproduce in the conditions of oxygen limited supply are well known now [5,6,7,8,9,10,11,12]. Significant attention is paid to the risk of flooding to crops, which are mostly terrestrial plants sensitive to anaerobic soil conditions, which drastically reduce yields [13,14,15,16,17,18]. The impact of soil flooding on wild psammophytes—an ecological group, which is part of the ecological group xerophytes, adapted to arid conditions—has, until now, been mostly ignored.

Psammophytes grow in sandy soils on beaches, deserts, the edges of pine forests, and sand dunes. They exhibit varying degrees of species diversity depending on habitat, play an important role in soil stabilization and soil nutrient dynamics in sandy habitats, and are often considered extremophiles. *Alyssum desertorum* Stapf (desert beetroot, family Brassicaceae), the native range of which is Central Europe to Central Asia, is well adapted to arid conditions and soil of light and medium structure [19]. In Ukraine, it grows on dry slopes and outcrops of various rocks, on open slopes of mountains and hills, in flat steppes on sandy soils, and less often in pine forests and on their edges. In addition, it is ephemeral and dries up after fruiting [20]. *Secale sylvestre* Host (a wild species of rye, family Poaceae) grows on loose sandy and loamy soils from the Hungarian plain to Central Asia. In Ukraine, it is found in dense massifs in the steppe and forest-steppe zones [21].

To elucidate the morphological and biochemical responses and tolerance of these two psammophytes to soil flooding, we performed simulation flooding experiments. Since photosynthesis is very sensitive to the effects of drought and flooding [22,23,24], we investigated the effect of soil flooding on chloroplast ultrastructure and chlorophyll fluorescence induction. Plant adaptation to hypoxia conditions at the biochemical level is provided by enhanced anaerobic energy metabolism [4,25,26,27]. The activity of alcohol dehydrogenase (ADH) and pyruvate decarboxylase (PDC), which are key enzymes of ethanol fermentation, is considered one of the important indices of plant resistance to waterlogging [6,28,29,30,31]. The synthesis of heat shock proteins (HSP), which function as molecular chaperones and protect protein homeostasis [32,33], is also well known as the key component of cellular responses to a changing environment, in particular to hypoxia under waterlogging [34,35,36,37,38]. So, we also examined ADH as the key enzyme of anaerobic energy metabolism and HSP70 as a marker of stress reaction in the leaves of the experimental plants using biochemical methods.

## 2. Results

### 2.1. Ultrastructure of the Photosynthetic Apparatus

#### 2.1.1. Alyssum Desertorum

**Figure 1:**
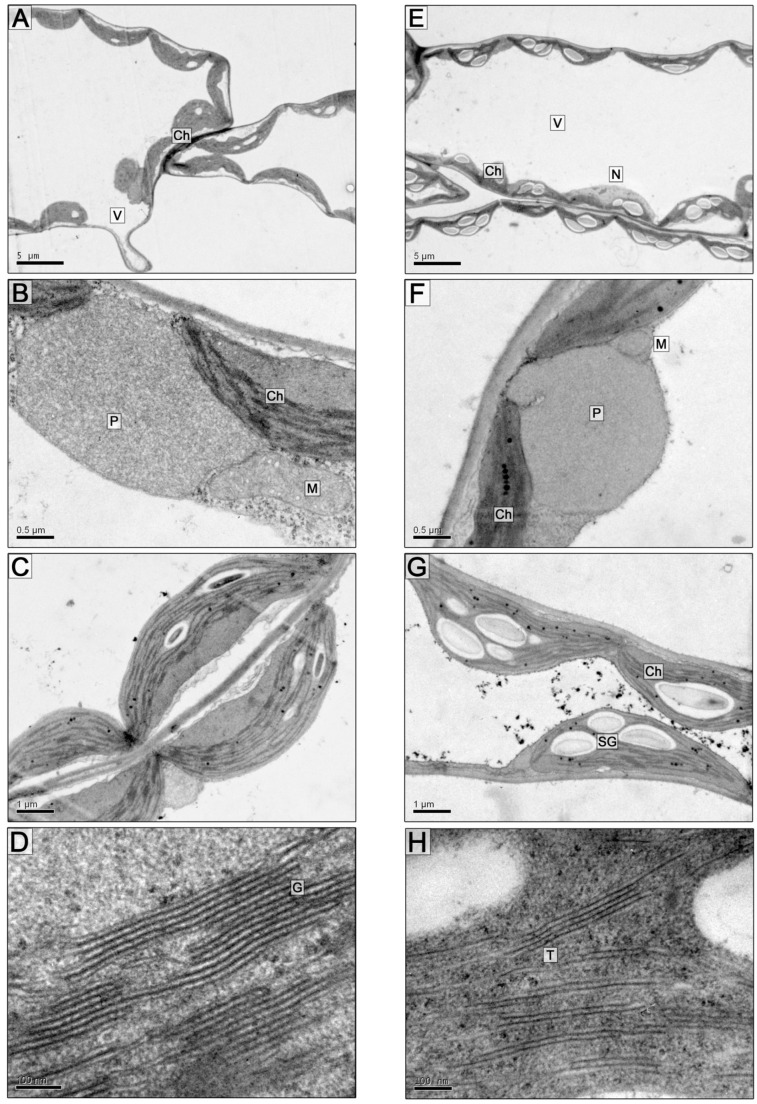
Cells (**A**,**E**) and cell fragments (**B**–**D**,**F**–**H**) of the *A. desertorum* leaf mesophyll. (**A**–**D**)—control, (**E**–**H**)—5 days of soil flooding. Scale bars: 5 µm (**A**,**E**), 0.5 µm (**B**,**F**), 1 µm (**C**,**G**), 100 nm (**D**,**H**). Abbreviations: Ch—chloroplast, T—thylakoid, G—granum, SG—starch grain, P—peroxisome, M—mitochondrion, N—nucleus, V—vacuole.

The leaves of *A. desertorum* plants growing in sandy soil in Ukraine are densely pubescent with stellate trichomes, and the leaf adaxial and abaxial surfaces are covered with a cuticle. The mesophyll is weakly differentiated into palisade and spongy parenchyma. In general, the leaf micromorphology is similar to that in plants of this species growing in Eastern Anatolia, Turkey [39]. The ultrastructure of the mesophyll cells is typical for photosynthesizing cells—a large central vacuole and a cytoplasm peripheral layer with organelles located in it (Figure 1A).

**Figure 2:**
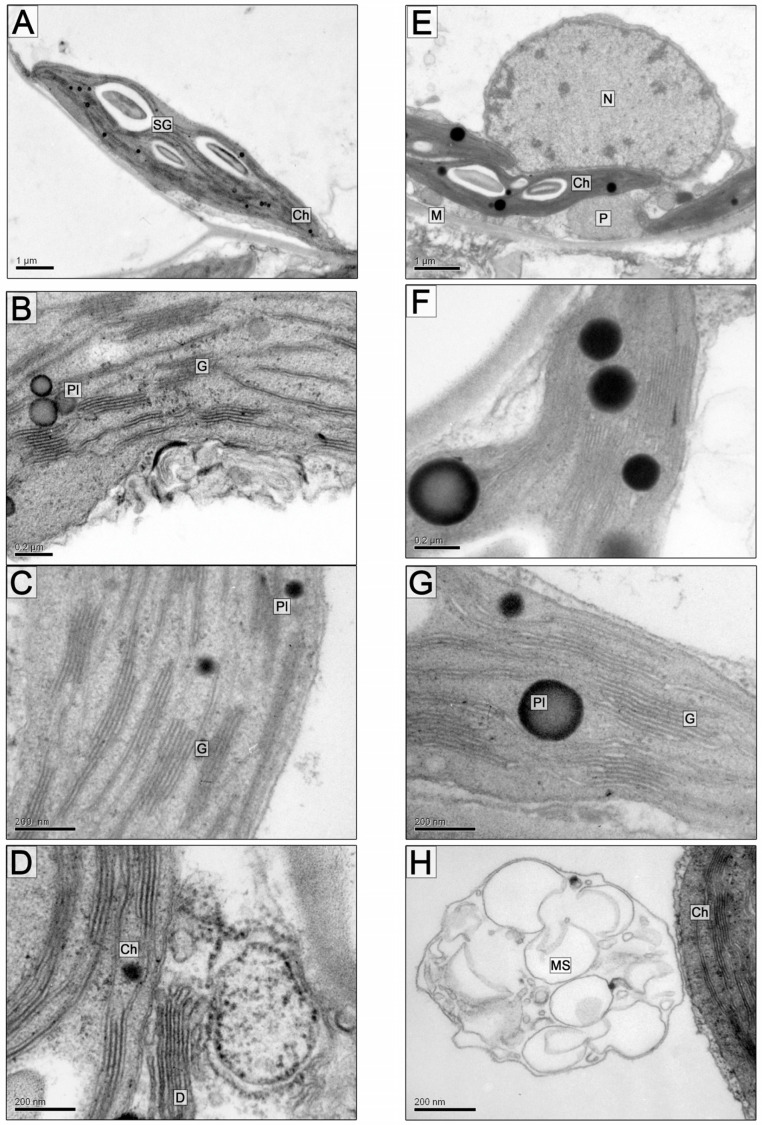
Fragments of the *A. desertorum* leaf mesophyll cells. (**A**–**D**)—control, (**E**–**H**)—10 days of soil flooding. Scale bar: 1 µm (**A**,**E**), 0.2 µm (**B**,**F**), 200 nm (**C**,**D**,**G**,**H**). Abbreviations: Ch—chloroplast, G—granum, SG—starch grain, Pl—plastoglobule, M—mitochondrion, P—peroxisome, D—dictyosome, N—nucleus, MS—multivesicular structure.

Chloroplasts, depending on the cut plane, had an elongated, oval, or rounded shape and were in close contact with mitochondria and single large peroxisomes, on average 2.29 ± 0.14 μm in diameter (Figure 1B; Table 1) with a granular and thin fibrillar content of medium electron density, sometimes with electron-dense clusters of granular material on the periphery of organelles in the contact zones between the mitochondria and plastids. Particularly large, over 3 μm in diameter, peroxisomes were observed in cells that surrounded the vascular bundles. Plastids contained starch grains (Figure 1C and Figure 2A,B) and plastoglobuli (Figure 2B,C), an average of 15 per organelle. The chloroplast grana consisted of 6.5 thylakoids on average (Figure 1D and Figure 2B,C, Table 1).

The population of mitochondria with well-developed cristae is polymorphic; round, oval, and elongated organelles are observed.

On the 5th and 10th day of soil flooding, the general organization of mesophyll parenchyma cells is mainly similar to that in the control; there are also single large peroxisomes (Figure 1E,F). Certain differences were found in the ultrastructure of the chloroplasts; first of all, a significant, almost twofold, increase in the size of the starch grains was observed, especially on the 5th day of flooding (Figure 1G and Figure 2E; Table 1 and Table 2). The diameter of plastoglobuli on the 5th day of flooding was not significantly different from the control, but, on the 10th day, the plastoglobule diameter of the flood-treated samples was twice as large as in the control (205.32 ± 26.19 nm and 101.2 ± 12.46 nm, respectively: Figure 2F,G; Table 1).

The number of thylakoids in grana on the 5th day of the experiment did not differ significantly in the control and after flooding. On the 10th day, the number of thylakoids per granum increased slightly (11.65 ± 1.76) compared to the control (8.2 ± 0.9) (Figure 1H and Figure 2G; Table 2). Usually, granal thylakoids are densely packed, but under flooding, granal and stromal thylakoids with an increased lumen were sometimes observed (Figure 2G). On the 5th day of flooding, the linear dimensions of chloroplasts increased due to the augmentation of starch granules. On the 10th day of flooding, the length of plastids decreased slightly (Table 1), but their number increased, and in general, the organelles had smoothed, slightly rounded contours along with an increased starch content and enlarged plastoglobuli. During flooding, the size of the mitochondria in the sections did not change significantly (Table 1). Accumulations of multivesicular structures in the vacuole were observed more often, which can be considered indicative of increased autophagy of the cytoplasm under the influence of hypoxia (Figure 2D,H).

#### 2.1.2. Secale Sylvestre

**Figure 3:**
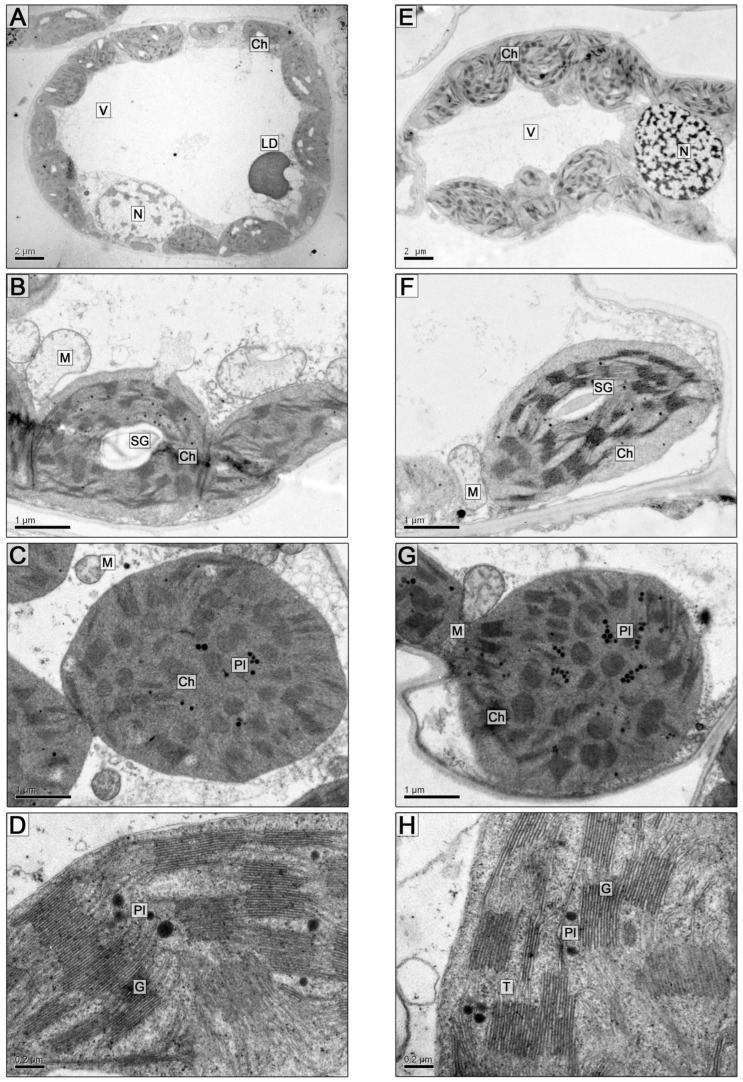
Cells (**A**,**E**) and cell fragments (**B**–**D**,**F**–**H**) of the *S. sylvestre* leaf mesophyll. (**A**–**D**)—control, (**E**–**H**)—5 days of soil flooding. Scale bar: 2 µm (**A**,**E**), 1 µm (**B**,**C**,**F**,**G**), 0.2 µm (**D**,**H**). Abbreviations: Ch—chloroplast, T—thylakoid, G—granum, SG—starch grain, Pl—plastoglobule, M—mitochondrion, N—nucleus, V—vacuole, LD—lipid droplet.

**Figure 4:**
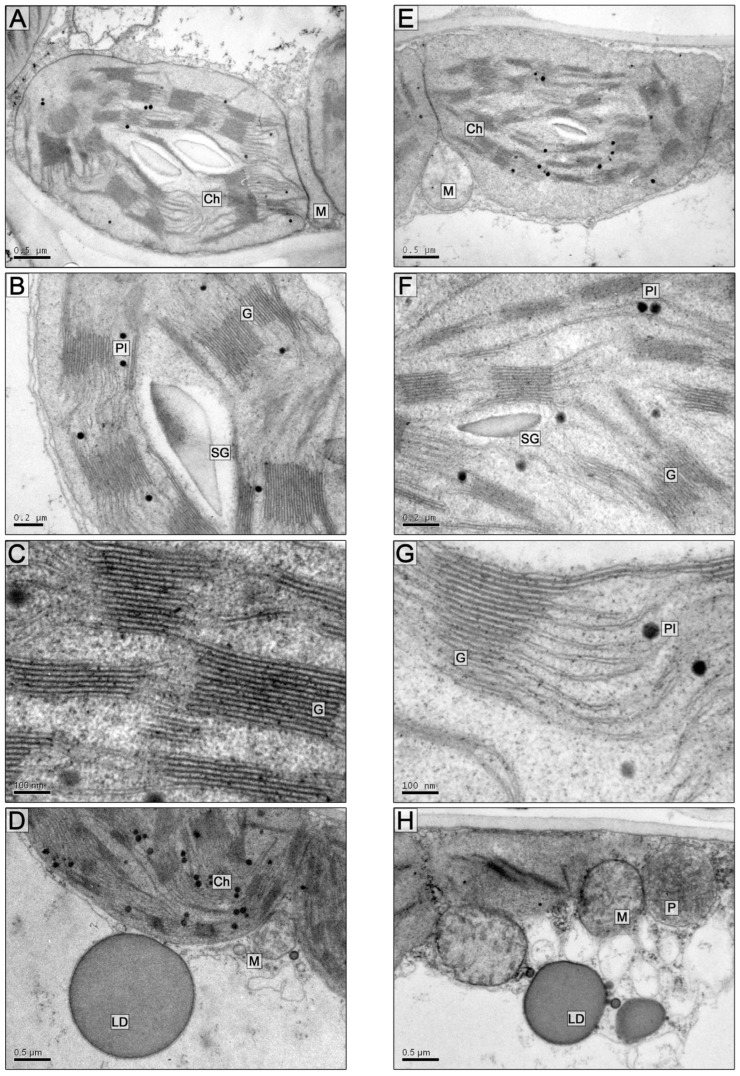
Fragments (**A**–**H**) of the *S. sylvestre* leaf mesophyll cells. (**A**–**D**)—control, (**E**–**H**)—10 days of soil flooding. Scale bars: 0.5 µm (**A**,**D**,**E**,**H**), 0.2 µm (**B**,**F**), 100 nm (**C**,**G**). Abbreviations: Ch—chloroplast, G—granum, SG—starch grain, Pl—plastoglobule, M—mitochondrion, P—peroxisome, LD—lipid droplet.

The leaves of *S. sylvestre* plants are covered with simple needle-like trichomes on both surfaces, isobilateral and amphistomatic. The ultrastructure of leaf mesophyll cells of *S. sylvestre* is typical for photosynthesizing cells—a large central vacuole and a cytoplasm peripheral layer with organelles located in it (Figure 3A). Most chloroplasts with starch grains had oval or rounded shapes (Figure 3A–C and Figure 4A,B) and were in close contact with mitochondria and peroxisomes, which sometimes contained electron-dense fibrils.

The population of mitochondria with moderately developed cristae is polymorphic: round, oval, and elongated organelles were observed. Single lipid droplets 1.5–2 μm in diameter were sometimes observed (Figure 4D).

The ultrastructural organization of mesophyll parenchyma cells on the 5th and 10th days of soil flooding was basically similar to the control. Differences were found in the size of starch grains, which decreased particularly on the 10th day of flooding (0.14 ± 0.03 µm^2^ and 0.46 ± 0.04 µm^2^ in the control: Figure 3E,F and Figure 4E,F; Table 3 and Table 4).

On the 10th day of flooding, linear dimensions of chloroplasts decreased, and round organelles were observed (Figure 4G; Table 3). The average number of thylakoids per granum did not differ significantly between the control (10.8 ± 0.92) and flooded chloroplasts (11.5 ± 1.25) on the 5th and 10th days of the experiment. On the 10th day, the partial volume of thylakoids (both granular and stromal) was greater in the control (21.4 ± 2.7 and 19.1 ± 2.2) compared to plastids from flooded samples (20.5 ± 2.5 and 17.2 ± 2.1). However, on the 5th day of flooding, the partial volume of thylakoids (20.9 and 16.6) exceeded the control values (15.6 ± 1.8 and 12.4 ± 1.5) (Figure 3C,D and Figure 4B; Table 4). The diameter of plastoglobules and their partial volume per chloroplast increased slightly (Table 3 and Table 4).

Under soil flooding, the size of mitochondria did not change significantly, but the matrix of organelles became more electron-lucent, which may indicate reduced respiratory activity. Lipid droplets decreased in size but increased in number (Figure 4H).

### 2.2. Chlorophyll Content

In *S. sylvestre*, a decrease of 45–59% in the content of chlorophylls and carotenoids in leaves compared to the control was observed on both the 5th and 10th day of soil flooding (Table 5). The chlorophyll *a*/*b* ratio increased after 5 days and decreased after 10 days of flooding. In *A. desertorum*, the content of photosynthetic pigments was less affected by flooding and decreased only by 5–25%. The most pronounced was the decrease in the content of chlorophyll *b*, which constituted 25% after 5 days and 15% after 10 days of the treatment. Therefore, soil flooding consistently increased the chlorophyll *a*/*b* ratio.

### 2.3. Chlorophyll Fluorescence Induction

**Figure 5:**
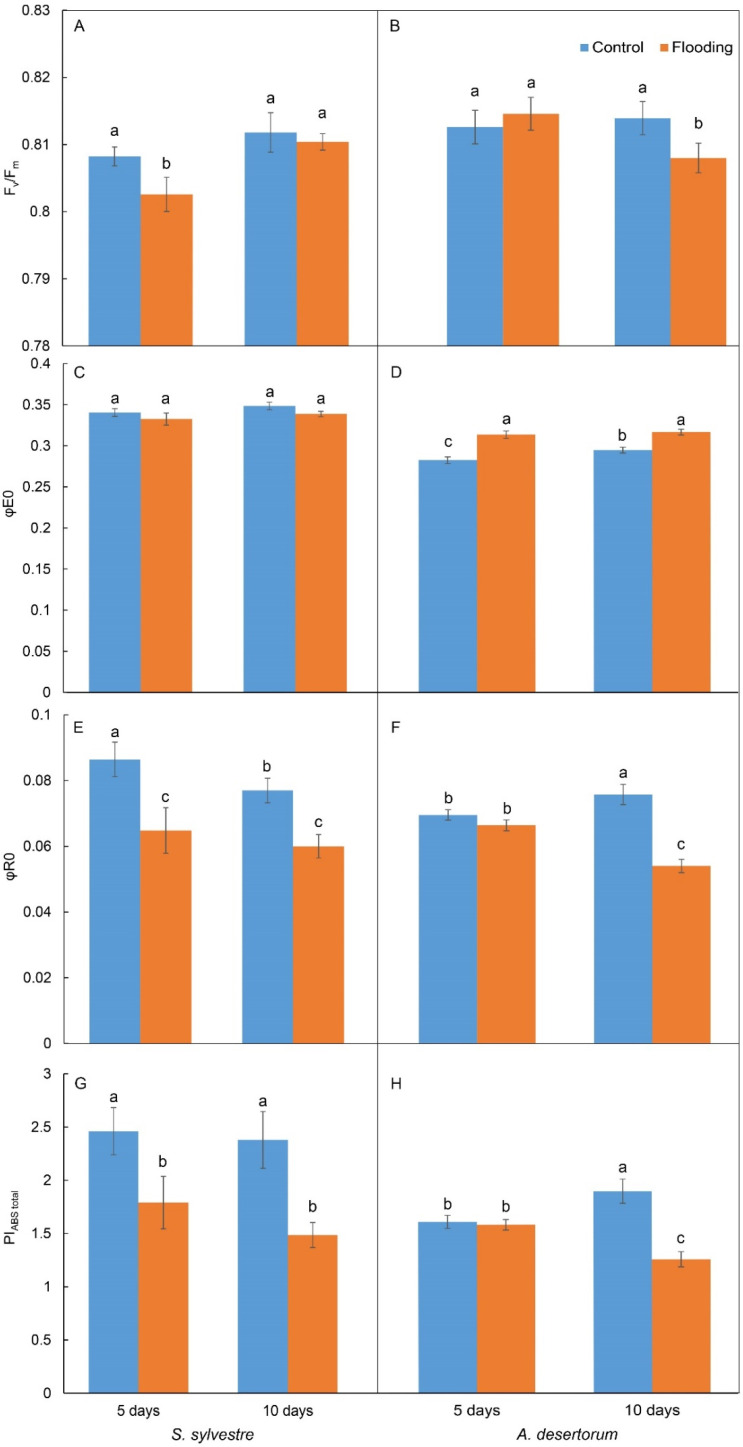
The effect of flooding on key parameters of JIP test in *S. sylvestre* and *A. desertorum*: F_v_/F_m_ (**A**,**B**), φE0 (**C**,**D**), φR0 (**E**,**F**), and PI_ABS,total_ (**G**,**H**) in leaves after 5 and 10 days of treatment. Error bars represent the standard error (S.E.) of mean (*n* ≥ 30). Different letters indicate significant differences at *p* < 0.05.

The observed values of F_v_/F_m_ (Figure 5A,B) were generally higher than 0.8, indicating little evidence of stress in all variants, although flooding affected this parameter. In *S. sylvestris*, a decrease in F_v_/F_m_ was observed only after 5 days of treatment, and after 10 days, the difference was insignificant (Figure 5A). φE0 was not affected by flooding (Figure 5C), while both φR0 and PI_ABS,total_ were decreased significantly to a similar extent at both 5 and 10 days of the experiment (Figure 5E,G), suggesting decreased efficiency of the total linear electron transport in chloroplasts due to the limitation of electron transfer from photosystem I to NADP^+^.

In *A. desertorum*, the negative effect of flooding was more pronounced after 10 days of treatment, and φE0 was even slightly stimulated (Figure 5D). The level of PS 2 damage was generally lower than that in *S. sylvestris*, and on day 10, it was slightly lower in the control than on day 5. However, a pronounced negative effect of flooding was observed only after 10 days. The integral indicators of the efficiency of photosynthesis (φR0 and PI_ABS,total_) decreased significantly only on the 10th day of the experiment.

### 2.4. Protein Spectrum, HSP70, and Alcohol Dehydrogenase Synthesis in Leaves

**Figure 6:**
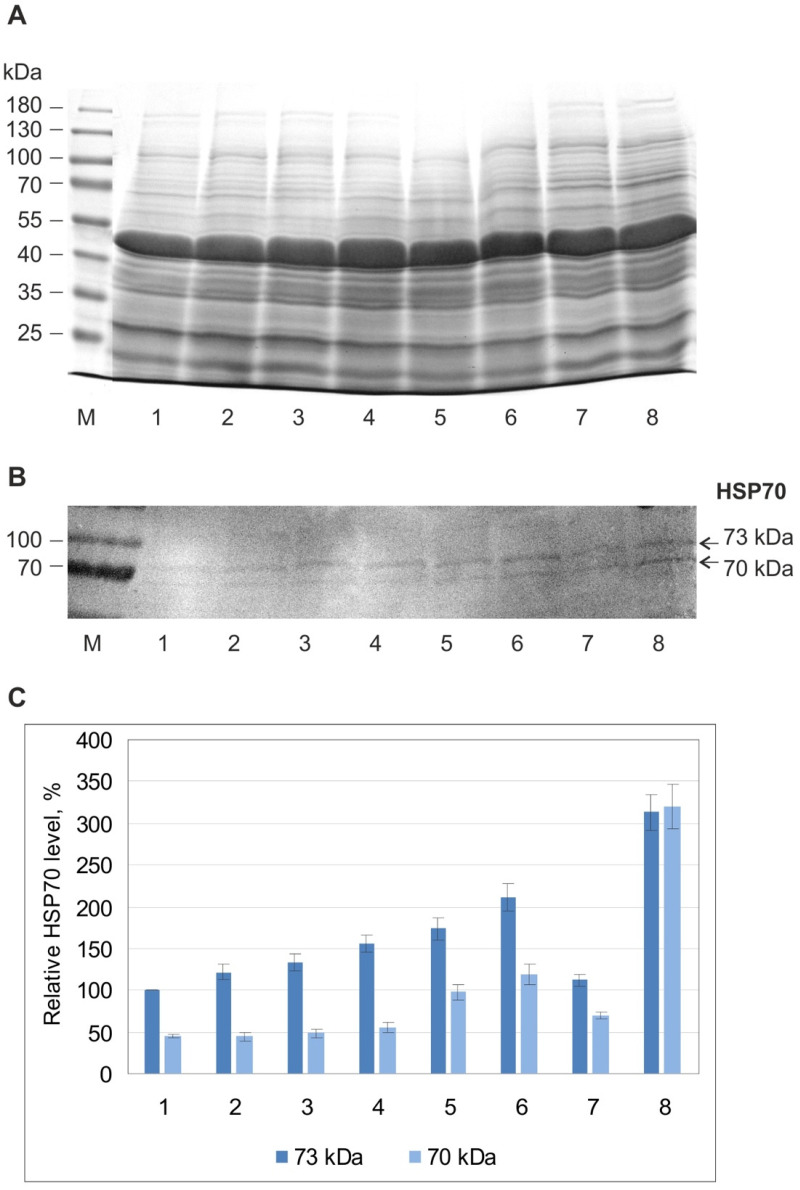
Effect of flooding on protein spectrum (**A**) and HSP70 (**B**,**C**) in leaves of Alyssum desertorum plants. (A) 10% SDS-PAG electrophoretic pattern of soluble proteins, (**B**) Western blot of HSP70, and (**C**) the results of densitometric analysis of the Western blots. (1) Control 1 (terrestrial plants before flooding), (2–6) under flooding for 4 h (2), 1 day (3), 2 days (4), 4 days (5), 6 days (6), (7) control 2 (terrestrial plants after 6 days of the experiment), (8) 40 °C for 2 h (internal control for HSP70 induction). (M) Molecular weights of marker proteins. In diagram (**C**), the relative HSP70 levels are expressed as the percent difference from the basal level of the 73 kDa protein in control 1 (100%). The data are the means and standard deviations from three independent experiments.

**Figure 7:**
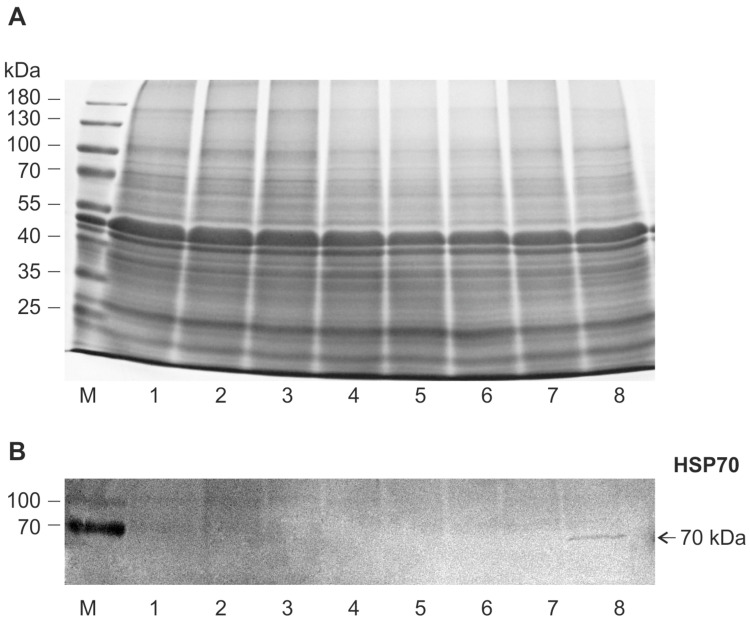
Effect of flooding on protein spectrum (**A**) and HSP70 (**B**) in leaves of *Secale sylvestre* plant. (**A**) 10% SDS-PAG electrophoretic pattern of soluble proteins, (**B**) Western blot of HSP70: (1) control 1 (terrestrial plants before flooding), (2–6) under flooding for 4 h (2), 1 day (3), 2 days (4), 4 days (5), 6 days (6), (7) control 2 (terrestrial plants after 6 d of the experiment), (8) 40 °C for 2 h (internal control for HSP70 induction). (M) Molecular weights of marker proteins.

SDS-PAG electrophoresis of the soluble protein of leaves did not reveal noticeable qualitative and quantitative changes in the protein spectrum in both psammophyte species during 6-day flooding (Figure 6A and Figure 7A). Western blot analysis of HSP70 identified two isoforms (70 kDa and 73 kDa) in *A. desertorum*, both of which showed a slight increase during flooding (Figure 6B). However, the degree of their increase was less compared to the heat shock reaction (Figure 6B, var. 8). In *S. sylvestre*, one HSP70 band was detected only in heat-shocked leaves and was not detectable under flooding (Figure 7B). The same result was obtained with plants grown from seeds collected in two different years.

**Figure 8:**
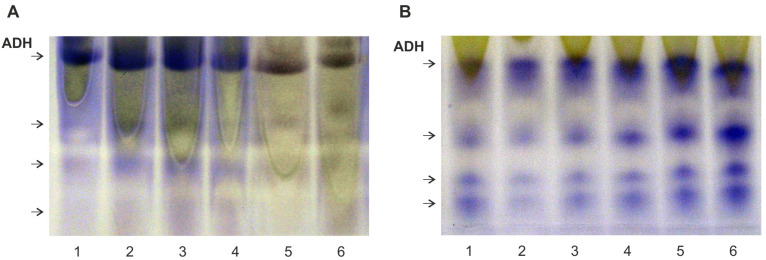
Effect of flooding on expression of ADH in leaves of *Alyssum desertorum* (**A**) and *Secale sylvestre* (**B**) plants. Native gel electrophoresis of the protein followed by staining for ADH activity was performed. (1) Control (terrestrial plants before flooding), (2–6) under flooding for 4 h (2), 1 day (3), 2 days (4), 4 days (5), 6 days (6).

To test ADH in leaves when the roots were flooded, native protein electrophoresis was used, followed by staining of the product of the enzymatic reaction in the gel. ADH zymograms of both plant species contained four bands with enzyme activity (Figure 8). The total intensity of their color in each variant reflects the ADH level. *A. desertorum* showed weak enzyme staining in the control, some activation of its synthesis for the first day of flooding, and a decrease after 4 days (Figure 8A). In contrast, in *S. sylvestre*, a gradual increase in ADH levels began after 1 day and reached a maximum by the 6th day (Figure 8B).

### 2.5. Ethylene Assay

**Figure 9:**
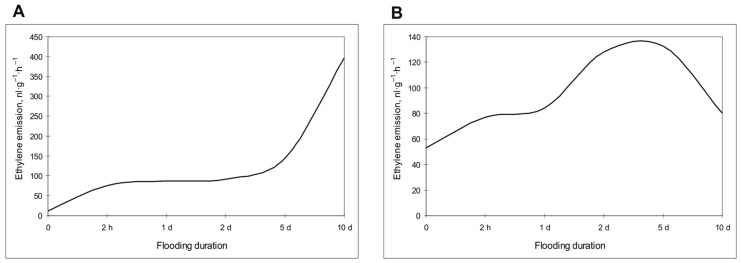
Effect of flooding on ethylene emission in *Alyssum desertorum* (**A**) and *Secale sylvestre* (**B**) plants. Ethylene emission is presented as nL·g^−1^ fresh weight·h^−1^. Smoothed plots of averages from 2 independent experiments (3 replicates each).

*A. desertorum* plants responded to flooding by emitting ethylene up to ~100 nL g^−1^ h^−1^ during the first 2 h, maintaining this level for 5 days, and then increasing it four times by the 10th day (Figure 9A). The response of *S. sylvestre* also started with a rapid increase in ethylene production to ~80 nL g^−1^ h^−1^, increasing almost twofold in 5 days, but then decreased by the 10th day (Figure 9B).

## 3. Discussion

### 3.1. Chloroplast Ultrastructure and the Content of Photosynthetic Pigments in Leaves

It is well known that soil flooding inhibits enzyme activity related to photosynthesis, resulting in decreased chlorophyll synthesis and a reduction in photosynthetic rate, leading to leaf yellowing, senescence, and death of the plants [11,40]. Yellowing of leaves is considered a visible symptom of flooded plants. Changes in the content of photosynthetic pigments and chloroplast ultrastructure in the investigated psammophytes under soil flooding are comparable with those of mesophytes—plants that grow in soil with medium moisture content—in the same conditions, namely: increased or decreased chloroplast size, chloroplast “rounding”, dilated thylakoids with swollen lumen, increased number and size of plastoglobuli, increased or decreased size of starch grains, emergence of stroma invaginations and protrusions, and decreased chlorophyll content. For example, decreasing chlorophyll and carotenoid contents were reported for mesophyte plants of *Momordica charantia* [41], *Hordeum vulgare* [42,43], *Triticum aestivum* [44,45], *Vicea faba* [46], and *Sesamum indicum* [17]. In *Zea mays* seedlings, after the waterlogging treatments for 3 and 6 days, significantly declined leaf chlorophyll content, decreased F_v_/F_m_ and *Φ*_PSII_, reduced numbers of grana and granal thylakoids, and chloroplasts of changed shape, as well as significantly decreased photosynthetic capacity, have been described [47,48]. Similar changes in chloroplast size and shape, and also swelled granal thylakoids under waterlogging, have been reported in *Kosteletzkya virginica* [49] and *Phoebe sheareri* [50]. The F_v_/F_m_ ratio, especially in young *V. faba* seedlings, was lower under flooding in comparison with the control [46]. The decreased photosynthetic pigment content, inhibited leaf carbon assimilation, and limited PSII electron transport efficiency have also been described in *Zingiber officinale* under waterlogging [51]. Modification of the structure of chloroplasts, in particular, condensation or swelling of granal thylakoids, is believed to affect the structure of photosystem I (PSI) and photosystem II (PSII) [22,52] and reduce maximum PSII quantum efficiency [53]. An increase in thylakoid lumen may facilitate the diffusion of plastocyanin, increasing the rate of electron transport between the two photosystems [49,54]. A model of control of electron transport/photoprotection is proposed, which requires a clear consideration of the ultrastructural dynamics of thylakoids, depending on the level of water exchange between the cytosol and chloroplasts in response to variations in environmental conditions, primarily light intensity [55].

The accumulation of transient starch in chloroplasts under waterlogging is reported in species sensitive to hypoxia, e.g., *Helianthus annuus* [56], *Citrus jambhiri* and *C. aurantium* [57], *Phoebe sheareri*, *Chionanthus virginicus*, and *Carya illnoinensis* [50], although it is also known for resistant species, e.g., *Quercus alba* [58], *Eucalyptus globulus* [59], and *Distylium chinense* [60], and is associated with the suppression of photosynthesis. Transient starch accumulates inside the chloroplasts in the daytime and at night is degraded into glucose and maltose, which are exported to the cytosol for sucrose synthesis or as energy sources. Then, sugars produced by photosynthesis are transported to other organs, in particular to roots, via the phloem [61,62]. The accumulation of transient starch in leaves, as well as soluble carbohydrates in the phloem and the low concentration of carbohydrates in the roots, is thought to be the result of a decrease in the rate of phloem transport to the roots caused by the inhibition of root aerobic metabolism under low oxygen conditions [53,56,63]. It has been suggested [64] that starch accumulation in chloroplasts, reduced leaf chlorophyll content, reduced activity of carboxylation enzymes, and maximum PSII quantum efficiency underlie a decrease in the photosynthetic capacity under flooding. At the same time, the reduction in starch in the leaf chloroplasts of various plant species in response to flooding, e.g., *Nicotiana tabacum* [65], as well as drought, salinity, or extreme temperature, which often correlates with increased plant resistance to the stressor, has been reported [66]. Therefore, starch metabolism becomes a key factor determining plant survival under adverse conditions, which requires further research to clarify the dependence on the plant species and ecology, organ, and tissue type and the nature of the active factor. Our data on the changes in transient starch volume in chloroplasts of *A. desertorum* and *S. sylvestre* in response to soil flooding clearly demonstrate the species-dependent nature of starch metabolism. Both species are psammophytes and grow in the same conditions but react differently to hypoxia according to this characteristic: in the first species, starch volume in chloroplasts increased, while in the second species, it decreased.

Plastoglobuli are supposed to maintain a constant lipid/protein ratio in thylakoid membranes through a dynamic exchange of lipids with membranes in organelle biogenesis, metabolism, developmental transitions, and responses to stress, providing fast adjustments to changing environments. It was reported that the size and number of plastoglobuli increase under unfavorable conditions [67,68]. Data about increasing the number and size of plastoglobuli in chloroplasts of *A. desertorum* and *S. sylvestre*, as well as *Kosteletzkya virginica* [49], *Phoebe sheareri*, *Chionanthus virginicus*, and *Carya illnoinensis* [50], under waterlogging fit logically into these ideas.

A decrease in chlorophyll content is considered a marker of impaired photosynthesis and damage to photosynthetic apparatus, which was usually observed under waterlogging conditions and accompanied by visual yellowing of leaf tips [46,47]. A decrease in photosynthetic pigment content is consistent with the results of the JIP test, where we observed a decrease in photosynthetic activity at the level of electron flow to the final acceptors (decrease in φR0 and PI_ABS,total_). Based on the results of the JIP test and the content of chlorophylls, it can be concluded that the photosynthesis of *S. sylvestre* is sensitive to soil flooding. The less pronounced effect of flooding on the content of photosynthetic pigments and the increased chlorophyll *a*/*b* ratio in *A. desertorum* is consistent with the results of the JIP test, which revealed an increase in the efficiency of electron transfer from photosystem 2 to plastoquinone and a decrease in total electron transport efficiency only after 10 days of the treatment. At least up to 10 days of soil flooding, the photosynthetic apparatus of *A. desertorum* is less sensitive to flooding than *S. sylvestre*.

### 3.2. Chlorophyll a Fluorescence Induction and JIP-Test

Flooding caused a significant decrease in total photosynthesis efficiency in both studied plants, but in *A. desertorum*, this effect was observed only after 10 days of the treatment, as indicated by decreased values of PI_ABS,total_. This effect may be caused by decreased stomatal conductance and limited CO_2_ diffusion to mesophyll cells that were reported under conditions of soil flooding (Pezeshki, 2001, as cited in [69]). Similar effects were shown in plants sensitive to waterlogging [46,47]. At the same time, the treatment did not lead to pronounced damage to the photosynthetic apparatus, as indicated by relatively high F_v_/F_m_ values in all variants that were only slightly decreased after flooding for 5 (in *S. sylvestre*) or 10 days (*A. desertorum*).

A slight increase in φE0 in *A. desertorum* indicates increased efficiency of electron transfer to the platoquinone pool from photosystem II. Although this effect may appear paradoxical, it can result from a decreased light-harvesting antennae size that is indicated by an increased chlorophyll *a*/*b* ratio. Decreased antennae lead to less quanta being absorbed per reaction center, lowering the Q_A_ reduction rate and excitation pressure [70]. Thus, such an increased efficiency of the partial electron transport is caused by decreased excitation pressure rather than facilitated electron transfer on the photosystem 2 acceptor side.

### 3.3. Role of HSP70 and ADH in Response to Flooding

The main effect of short-term waterlogging is O_2_ shortage (hypoxia), leading to energy deprivation in roots. In turn, root hypoxia causes a systemic response in a plant organism, including reprogramming protein synthesis, stress response, and anaerobic adaptation.

Electrophoretic analysis of the total protein spectrum in leaves of *A. desertorum* and *S. sylvestre* clearly showed that both species are able to maintain normal protein composition at least for the first days of soil flooding. At the same time, analysis of HSP70 as an indicator of stress response and ADH as an indicator of anaerobic adaptation showed the response of protective and adaptive systems and revealed differences between species.

Molecular chaperones/HSPs protect cellular proteostasis under stressful conditions [32,71]. Their accumulation enhances the tolerance of plant organisms to environmental variations. Inducible members of the HSP70 family are considered major actors of the stress response in many species including plants [32,72]. A certain upregulation of two HSP70 isoforms (70 kDa and 73 kDa) in leaves of *A. desertorum* in response to soil flooding showed a weak systemic response in this species. This is consistent with our previous results obtained for mesophyte *Malva* and hydrophyte *Sium sisaroideum,* where soil flooding led to a significant upregulation of HSP70 in the leaves [37,73]. In contrast, one HSP70 isoform (70 kDa) in *S. sylvestre* was detected only at heat shock and was not detected during flooding. Given the significant changes in other traits in plants of this species, it is difficult to assume that flooding does not cause a stress response in this case. Therefore, we may suppose that other chaperones and/or protective systems play a key role in maintaining proteostasis in this species.

Metabolic adaptation of plants to oxygen shortage is provided by the fermentative pathway that consists of two steps: pyruvate decarboxylase (PDC) catalyzes the decarboxylation of pyruvate to acetaldehyde and ADH catalyzes the subsequent reduction of acetaldehyde to ethanol with concomitant oxidation of NAD(P)H to NAD(P)^+^ [6,28,29,74]. The ADH function is inherent in hydrophytes, allowing them to withstand hypoxia. The presence of *ADH* genes in mesophytes and xerophytes and their upregulation, to some degree, in response to flooding conditions have been repeatedly reported [3,11,26,75,76]. Thus, the induction of the *ADH1* gene in seedling roots of *Coix lacroyma-jobi* after soil flooding was shown and it reached the highest level after 4 h [27]. In the allotetraploid *Gossypium hirsutum*, which is highly sensitive to waterlogging, there are three ADH isozymes, and ADH activity increased several-fold in both the roots and shoots of seedlings after flooding [77]. *ADH1* and *ADH2* expression increased rapidly in the roots of *Zea mays* seedlings after 4 h of anaerobic conditions through rigorous exclusion of O_2_, but it was followed by a rapid decline between 12 and 18 h [78]. In *Cucumis sativus* seedlings, *ADH* expression began in 2 h under soil flooding, reached a maximum at 4 h, and gradually declined after 8 h of flooding [79]. *ADH* expression and protein synthesis temporarily increased in seedling root tips of *Glycine max* after flooding [80]. Of at least six *ADH* genes in this species, *ADH2* expression increased most significantly after 6 h of flooding [81]. In *Hordeum vulgare*, the activity of *ADH1* could be detected during aerobic growth, and hypoxia induced the expression of *ADH1*, *ADH2*, and *ADH3* [82,83]. A significant increase in ADH level was reported in *Passiflora edulis* (var. *Flavicarpa*) seedlings in anaerobic conditions compared to seedlings under normal irrigation [84]. Soil flooding induced ADH synthesis in the desert species *Acacia erioloba*’s seedlings [85]. In the authors’ opinion, the presence of functionally active ADH genes in the desert plant is not clear and is probably connected with the limitation of oxygen supply during seed germination due to the hard seed coat. In *Arabidopsis thaliana*, which is highly sensitive to hypoxia, soil flooding caused increasing *ADH* expression in the first 6–8 h of flooding [34] with a protein maximum on the 6th day of flooding and a subsequent reduction that preceded plant death [86]. On the contrary, in *Sium sisaroideum*, which is intolerant to hypoxia, ADH synthesis was maintained at a high level during 10-day flooding accompanied by the formation of adventitious roots with aerenchyma [86,87]. The results of this research showed that psammophytes *A. desertorum* and *S. sylvestre* also have an ADH system and are capable of short-term anaerobic metabolic adaptation. Activation of ADH synthesis in leaves in response to root hypoxia indicated a systemic response. *S. sylvestre* showed a gradual activation of ADH synthesis with a maximum at the 6th day of soil flooding, which is similar to the dynamics, for example, in *A. thaliana* [86]. In *A. desertorum*, a weak rapid activation of ADH was detected for the first day of flooding followed by a gradual decrease, which was similar to, for example, the ADH expression pattern in *Cucumis sativus* [79]. Further flooding led to a decrease in the content of this protein, which preceded the death of the plants. These data suggest that *S. sylvestre* can gradually adapt to short-term soil flooding, while *A. desertorum* can only withstand impermanent flooding.

### 3.4. Ethylene Production in Response to Flooding

Another metabolic response to flooding is the production of the gaseous hormone ethylene, an important player in root-to-shoot signaling during the first hours of soil flooding [88]. The signal is the precursor of ethylene, 1-aminocyclopropane-1-carboxilic acid (ACC), which is converted to ethylene by ACC oxidase (ACO). It leads to faster rates of ethylene production in the aerial part of the plant. Ethylene can reduce plant damage through epinastic leaf curvature, fast stem growth, adventitious root, and aerenchyma formation [89,90]. In addition, ethylene can potentiate senescence [91].

A comparative analysis of ethylene production in leaves of two psammophyte species during flooding showed similar primary activation of ethylene emission in the first hours of flooding, but the subsequent dynamics were different. In *A. desertorum*, ethylene emission significantly enhanced after 5 days. In contrast, in *S. sylvestre*, the activation of hormone production was two times less and decreased by the 10th day. These data suggest that rapid activation of ethylene production allows plants to minimize the risk of damage and optimize plant growth during the early period of exposure. However, considering that both species are unable to adapt to long-term flooding, the data may also point to a role for ethylene as a signal for reduced growth and leaf senescence upon prolonged exposure [91,92]. In turn, species-specific differences in dynamics may be a consequence of different tuning of the signaling cascade, namely receptors (ETR), ion transporters (EIN2), and ethylene-responsive factors (ERF) [90].

## 4. Conclusions

Based on the obtained data, we conclude that photosynthesis or the photosynthetic apparatus of the investigated psammophytes functions during short-term soil flooding. The following decrease in the chlorophyll content and random ADH and HSP70 synthesis patterns indicate the absence of systemic anaerobic metabolic adaptation to long-term root hypoxia, leading to plant death. In our opinion, these results clearly demonstrate that adaptive phenotypic plasticity is the norm of the genotypes’ response to changing environments [93,94]. Thus, to improve the hypoxic tolerance of psammophytes, it is necessary to use the approaches and methods of genetic engineering.

## 5. Material and Methods

### 5.1. Plant Material

Seeds of *Alyssum desertorum* and caryopses of *Secale sylvestre* were collected from plants growing on the dry sandy areas of the ravine forests in the steppe zone of the Dnipropetrovsk region (*A. desertorum* 48.438965, 35.121684 and *S. sylvestre* 48.505838, 34.973898), sown in pots, and watered 5–10 mm above the soil surface until the *A. desertorum* seedlings showed four true leaves and the *S. sylvestre* seedlings showed tree leaves. The plants were grown at 22 ± 4 °C, with a 16 h light/8 h dark cycle, and a photosynthetic photon flux density of 100 ± 20 µmol quanta·m^−2^·s^−1^. Samples were withdrawn after 5 and 10 days of flooding for data measurement.

### 5.2. Transmission Electron Microscopy

Specimen cutoffs of 3 mm diameter were fixed in 3% glutaraldehyde (0.1 M cacodylic buffer, pH 7.2) for 3 h at ambient temperature and then in 1% osmium tetraoxide in the same buffer for 1 h at ambient temperature and 12 h at 4 °C. Samples were dehydrated through a graded acetone series and embedded in Epon–Araldite resins. Sections were obtained on an ultramicrotome PowerTome XL (Boeckeler Instruments, Tucson, AZ, USA). Ultrathin sections (about 55 nm) were stained with uranyl acetate and lead citrate and examined with a transmission electron microscope JEM 1230EX (JEOL, Tokyo, Japan).

### 5.3. Quantification of Leaf Blade and Chloroplast Structure

The thickness of leaf blades and palisade and spongy parenchyma and the size of chloroplasts from palisade parenchyma cells of young and mature leaves were determined from TEM micrographs with the UTHSCSA Image Tool 3.0 for Windows. The number of thylakoids in grana in chloroplasts in leaf cross-sections was counted.

### 5.4. Statistical Analysis

For statistical analysis of significance, quantitative data were analyzed by one-way ANOVA for 4 groups.

### 5.5. Pigment Analysis

The content of chlorophyll a and b and carotenoids was determined on the spectrophotometer UV1100 (Spectrolab, Shanghai, China). A total of 20 mg of crushed leaves was extracted in 7 mL of pure dimethyl sulfoxide. Extraction and measurements were performed according to [95,96]. A minimum of 5 biological replicates were performed for each variant.

### 5.6. Chlorophyll a Fluorescence Induction and JIP-Test

The state of the leaf photosynthetic apparatus was assessed with a custom-made portable OJIP fluorometer “G-rep” (Ihor Panas, Kyiv, Ukraine). This device can register the polyphasic fluorescence induction curve caused by the illumination of photosynthetic samples by a flash of high-intensity (saturating) exciting light. The intensity of this light in our experimental setup was 5000 µmol photons·m^−2^·s^−1^. The multiple (O, J, I, P) steps of this fluorescence rise are clearly visible on the logarithmic time axis and reflect the gradual reduction in electron carriers along the photosynthetic electron transport chain. By analyzing the parameters of this curve, it is possible to determine some traits of both light and dark phases of photosynthesis. The shape of the OJIP curve is sensitive to changes in photosynthesis caused by the environment. For the integral assessment of the photosynthetic apparatus state, the instrument’s sensor was pressed against the leaf blade after 15 min dark adaptation, and fluorescence changes were recorded for 1 s. Based on the obtained fluorescence curves, three quantum yields of electron fluxes and an integral index were calculated and analyzed.

1. φPo = F_V_/F_M_ is the maximum quantum yield of the primary photochemical reaction (at t_0_ = 0), which characterizes the probability of energy capture of the absorbed photons (or excitons migrating by the antenna) by the reaction centers of PS 2. In the case of a stress state, φPo is usually decreased.
2. φEo—quantum yield of electron transfer from PS 2 to plastoquinone.
3. φRo—quantum yield of reduction in electron terminal acceptors in the acceptor site of PS 1.
4. PI_ABS,total_—total performance index on an absorption basis, which characterizes the total function of the linear electron transport.

All calculations were performed according to [97].

### 5.7. Alcohol Dehydrogenase Analysis

To determine alcohol dehydrogenase (alcohol/NAD oxidoreductase, ADH, EC 1.1.1.1) level, native electrophoresis and ADH product staining were performed. Leaves of terrestrial plants before flooding (control) and plants flooded for 4 h, 1, 2, 4, and 6 days were used. A total of 0.3 g of leaf material was ground in a mortar with liquid nitrogen, homogenized with extraction buffer (0.1 M Tris-HCl, pH 7.0, 10% glycerol, 0.5% DTT, 1% Triton X100), and centrifuged at 5000 rpm and 4 °C for 5 min. An equal protein quantity of each sample was separated in 6% polyacrylamide gel (PAG) by native electrophoresis. For ADH staining, PAG was incubated in 1 M Tris-HCl buffer, pH 8.0, containing 10 mM NAD, 10 mM nitroblue tetrazolium (NBT), 10 mM phenazine methosulphate (PMS), and 0.6% ethanol. Three biological replicates were conducted.

### 5.8. Protein Extraction and Western Blot Analysis

Leaves of terrestrial plants before flooding (control 1), plants flooded for 4 h, 1, 2, 4, and 6 days, and terrestrial plants after 6 days of the experiment (control 2) were used. In addition, terrestrial plants heat shocked at 40 °C for 2 h were used as an internal control for HSP70 induction. A total of 0.3 g of leaf material was ground in a mortar with liquid nitrogen, homogenized with extraction buffer (25 mM Tris-HCl, pH 8.0, 20 mM NaCl, 1 mM EDTA, 1 mM protease inhibitor PMSF), and centrifuged at 12,000× *g* and 4 °C for 15 min. Protein concentration in the supernatant was determined according to the method in [98]. SDS buffer (0.125 M Tris-HCl, pH 6.8, 4% SDS, 20% glycerol, 5% β-mercaptoethanol) was added to the supernatant (1:1). Protein electrophoresis and Western blot analysis were conducted as described earlier [99]. An equal protein quantity of each sample was separated in 10% PAG-SDS. After electrophoresis, a gel was either stained with Coomassie G-250 or used for Western blotting. Blots were photographed and band intensity was determined using GelAnalyzer 2019.1 (http://www.gelanalyzer.com/ accessed on 1 January 2024). HSP70 staining density in each blot was normalized to the 73 kDa protein in control 1 (100%). The PageRuler Prestained Protein Ladder 10–180 kDa (TermoFisher, Rodano, Italy) was used to determine the molecular weight of the proteins. Three biological replicates were conducted.

### 5.9. Ethylene Assay

Ethylene emission was evaluated according to the method of [100] with modifications [92]. Leaves of terrestrial plants of both species before flooding (control) and plants flooded for 2 h, 1, 2, 5, and 10 days were used. Freshly harvested leaf samples were incubated in 30 mL glass vials sealed with a rubber stopper for 24 h at 21 ± 1 °C in the dark. Then, 1 mL of gas was sampled from each vial, and the ethylene content was measured using a FOCUS GC gas chromatograph (Thermo Scientific, Rodano, Italy) with a flame ionization detector, a stainless-steel matrix 80/100 column PROPAC R (Sigma-Aldrich, Burlington, MA, USA), helium as a carrier gas, a column temperature of 90 °C, an injector temperature 110 °C, and a detector temperature 150 °C. Amounts of ethylene were expressed in nanoliters per gram of fresh tissue per hour (nL∙g^−1^ fresh weight∙h^−1^). Calibration was performed with an ethylene standard (Sigma-Aldrich, Erlangen, Germany).

## Figures and Tables

**Table 1:** Morphometric parameters of *Alyssum desertorum* palisade parenchyma cells in control and after 5 and 10 days of soil flooding.

Parameter/Variant	5 Days		10 Days	
	Control	Flooding	Control	Flooding
chloroplasts: length, µm width, µm	7.14 ± 0.55 ^c^ 2.85 ± 0.19 _b_	10.42 ± 0.8 ^a^ 2.88 ± 0.19 ^b^	8.26 ± 0.63 ^b^ 2.03 ± 0.13 ^c^	6.59 ± 0.56 ^c^ 3.39 ± 0.23 ^a^
starch grains surface, µm^2^	0.64 ± 0.03 ^c^	1.12 ± 0.08 ^a^	0.44 ± 0.04 ^d^	0.73 ± 0.06 ^b^
plastoglobule diameter, nm	57.06 ± 4.41 ^c^	59.73 ± 7.35 ^c^	101.2 ± 12.46 ^b^	205.32 ± 26.19 ^a^
thylakoids per granum, *n*	4.7 ± 0.53 ^c^	4.3 ± 0.46 ^c^	8.2 ± 0.9 ^b^	11.65 ± 1.76 ^a^
mitochondria diameter, µm	0.95 ± 0.06 ^a^	0.89 ± 0.02 ^a^	0.83 ± 0.04 ^d^	0.71 ± 0.04 ^c^
peroxisome diameter, µm	2.29 ± 0.14 ^b^	2.61 ± 0.17 ^a^	1.95 ± 0.07 ^c^	2.19 ± 0.12 ^b^

Note: there is no significant difference between the values of the parameters in rows with the same letters in superscripts at *p* ≤ 0.05, *n* = 50.

**Table 2:** Partial volumes of chloroplast components in *Alyssum desertorum* palisade parenchyma cells in control and after 5 and 10 days of soil flooding, %.

Control, Flooding/Days		Granal Thylakoids	Stroma	Stromal Thylakoids	Starch Grains	Plastoglobuli
5 days	control	22.8 ± 2.7 ^a^	47.4 ± 5.8 ^a^	18.1 ± 2.1 ^a^	8.8 ± 0.7 ^b^	2.7 ± 0.2 ^b^
	flooding	21.2 ± 2.5 ^a^	45.5 ± 5.6 ^a^	16.9 ± 1.8 ^a^	13.4 ± 1.6 ^a^	2.9 ± 0.3 ^b^
10 days	control	19.2 ± 2.2 ^a^	51.5 ± 6.7 ^a^	17.2 ± 1.8 ^a^	9.0 ± 0.8 ^b^	2.8 ± 0.2 ^b^
	flooding	18.9 ± 2.3 ^a^	48.2 ± 6.2 ^a^	15.9 ± 1.6 ^a^	12.7 ± 1.4 ^a^	4.1 ± 0.5 ^a^

Note: there is no significant difference between the values of the parameters in rows with the same letters in superscripts at *p* ≤ 0.05, *n* = 50.

**Table 3:** Morphometric parameters of *Secale sylvestre* mesophyll cells in control and after 5 and 10 days of soil flooding.

Parameter/Variant	5 Days		10 Days	
	Control	Flooding	Control	Flooding
chloroplasts: length, µm width, µm	5.87 ± 0.42 ^a^ 2.74 ± 0.17 ^a^	5.02 ± 0.36 ^a^ 2.53 ± 0.16 ^a^	4.99 ± 0.38 ^a^ 2.18 ± 0.19 ^b^	4.14 ± 0.32 ^b^ 2.27 ± 0.22 ^b^
starch grain surface, µm^2^	0.19 ± 0.02 ^b^	0.13 ± 0.08 ^b^	0.46 ± 0.04 ^a^	0.14 ± 0,03 ^b^
plastoglobule diameter, nm.	42.5 ± 4.46 ^b^	53.7 ± 5.19 ^a^	50.3 ± 4.18 ^a^	61.4 ± 6.85 ^a^
thylakoids per granum, *n*	11.1 ± 0.85 ^a^	11.6 ± 1.28 ^a^	10.8 ± 0.92 ^a^	11.5 ± 1.25 ^a^
mitochondria diameter, µm	0.63 ± 0.04 ^a^	0.7 ± 0,02 ^a^	0.72 ± 0.04 ^a^	0.71 ± 0.04 ^a^
peroxisome diameter, µm	1.15 ± 0.09 ^a^	0.94 ± 0.06 ^b^	1.13 ± 0.07 ^a^	0.92 ± 0.07 ^b^

Note: there is no significant difference between the values of the parameters in rows with the same letters in superscripts at *p* ≤ 0.05, *n* = 50.

**Table 4:** Partial volumes of chloroplast components in *Secale sylvestre* mesophyll cells in control and after 5 and 10 days of soil flooding, %.

Control, Flooding/Days		Granal Thylakoids	Stroma	Stromal Thylakoids	Starch Grains	Plastoglobuli
5 days	control	15.6 ± 1.8 ^b^	69.1 ± 8.5 ^a^	12.4 ± 1.5 ^b^	1.6 ± 0.2 ^b^	1.3 ± 0.2 ^a^
	flooding	20.9 ± 2.5 ^a^	59.7 ± 7.3 ^a^	16.6 ± 1.9 ^a^	1.4 ± 0.2 ^b^	1.4 ± 0.2 ^a^
10 days	control	21.4 ± 2.7 ^a^	53.6 ± 6.5 ^a^	19.1 ± 2.2 ^a^	4.6 ± 0.8 ^a^	1.3 ± 0.2 ^a^
	flooding	20.5 ± 2.5 ^a^	59.1 ± 7.2 ^a^	17.2 ± 2.1 ^a^	1.8 ± 0.3 ^b^	1.4 ± 0.2 ^a^

Note: there is no significant difference between the values of the parameters in rows with the same letters in superscripts at *p* ≤ 0.05, *n* = 50.

**Table 5:** The effect of flooding on the content of photosynthetic pigments in *Secale sylvestre* and *Alyssum desertorum*.

Parameter	5 Days		10 Days	
	Control	Flooded	Control	Flooded
		*S. sylvestre*		
Chlorophyll *a* *	13.09 ± 0.25 ^b^	6.03 ± 0.20 ^d^	13.98 ± 0.20 ^a^	6.79 ± 0.12 ^c^
Chlorophyll *b*	4.45 ± 0.14 ^a^	1.85 ± 0.12 ^c^	4.24 ± 0.07 ^a^	2.37 ± 0.11 ^b^
Carotenoids	2.60 ± 0.09 ^a^	1.44 ± 0.17 ^b^	2.71 ± 0.08 ^a^	1.36 ± 0.07 ^b^
Chlorophyll *a*/*b* ratio	2.94	3.26	3.30	2.86
*A. desertorum*				
Chlorophyll *a*	7.22 ± 0.11 ^a^	5.67 ± 0.10 ^c^	7.00 ± 0.10 ^a^	6.68 ± 0.06 ^b^
Chlorophyll *b*	2.14 ± 0.06 ^c^	1.60 ± 0.06 ^d^	2.81 ± 0.04 ^a^	2.40 ± 0.05 ^b^
Carotenoids	1.52 ± 0.02 ^a^	1.23 ± 0.07 ^b^	1.47 ± 0.02 ^a^	1.37 ± 0.03 ^b^
Chlorophyll *a*/*b* ratio	3.38	3.54	2.49	2.78

*—concentrations of chlorophylls and carotenoids are presented as mg·g^−1^ dry weight. Note: there is no significant difference between the values of the parameters in rows with the same letters in superscripts at *p* ≤ 0.05, *n* = 5.

## Data Availability

The original contributions presented in the study are included in the article, further inquiries can be directed to the corresponding author.
